# Systematic Review and Meta-Analysis of Myocarditis Prevalence and Diagnostics in COVID-19:Acute, Post-COVID, and MIS-C (2020–2025)

**DOI:** 10.3390/jcm14197008

**Published:** 2025-10-03

**Authors:** Ioana-Georgiana Cotet, Diana-Maria Mateescu, Adrian-Cosmin Ilie, Cristina Guse, Ana-Maria Pah, Marius Badalica-Petrescu, Stela Iurciuc, Maria-Laura Craciun, Florina Buleu, Cristina Tudoran

**Affiliations:** 1 Doctoral School, Department of General Medicine, “Victor Babeş” University of Medicine and Pharmacy, Eftimie Murgu Square 2, 300041 Timisoara, Romania; ioana.cotet@umft.ro (I.-G.C.); diana.mateescu@umft.ro (D.-M.M.); cristina.marin@umft.ro (C.G.); 2 Department of Public Health and Sanitary Management, “Victor Babeş” University of Medicine and Pharmacy, Eftimie Murgu Square 2, 300041 Timisoara, Romania; ilie.adrian@umft.ro; 3 Cardiology Department, “Victor Babeş” University of Medicine and Pharmacy, Eftimie Murgu Square 2, 300041 Timisoara, Romania; marius.badalica-petrescu@umft.ro (M.B.-P.); iurciuc.stela@umft.ro (S.I.); laura.craciun@umft.ro (M.-L.C.); florina.buleu@umft.ro (F.B.); cristina.tudoran@umft.ro (C.T.)

**Keywords:** COVID-19, myocarditis, meta-analysis, cardiac magnetic resonance, MIS-C, vaccination, cardiac outcomes

## Abstract

**Background**: Myocarditis is a recognized complication of COVID-19, but prevalence estimates vary by disease phase and diagnostic method. **Methods**: We conducted a systematic review and meta-analysis of 54 studies including 32,500 patients, stratified by acute COVID-19, post-COVID, and MIS-C phases. **Results**: The pooled prevalence of myocarditis was 1.2% (95% CI: 0.8–1.6) in acute COVID-19, 7.4% (95% CI: 5.1–9.8) in post-COVID, and 39.8% (95% CI: 32.4–47.2) in MIS-C. CMR-based diagnosis yielded higher prevalence than clinical criteria (8.1% vs. 0.9%). Major cardiac outcomes included reduced LVEF in 22% and ventricular arrhythmias in 15% of cases. Heterogeneity across studies remained high (I^2^ = 98%). **Conclusions**: Myocarditis prevalence in COVID-19 varies widely across phases and diagnostic methods. Findings suggest a need for cautious screening approaches, particularly in MIS-C and selected post-COVID or athlete populations, while emphasizing the importance of standardized reporting and long-term follow-up data.

## 1. Introduction

Myocarditis, an inflammatory myocardial condition, can lead to ventricular arrhythmias, heart failure, and sudden cardiac death [1,2]. The pathophysiology involves direct viral invasion via ACE2 receptors, immune-mediated injury, cytokine storms, endothelial inflammation, and microvascular thrombosis [3,4,5,6,7]. Histopathology confirms myocardial inflammation, often with viral presence, even in asymptomatic cases [8,9,10]. The emergence of SARS-CoV-2 has highlighted myocarditis as a complication across acute COVID-19, post-COVID syndrome (defined as symptoms persisting ≥ 4 weeks post-infection per WHO guidelines [11]), and multisystem inflammatory syndrome in children (MIS-C) [12,13,14]. With over 700 million global COVID-19 cases and 7 million deaths by August 2025 [11,15], understanding cardiac complications is critical, particularly in low- and middle-income countries (LMICs) where diagnostic access, such as to cardiac magnetic resonance (CMR), is limited [16]. Studies report ventricular arrhythmias in up to 20% of COVID-19 myocarditis cases and reduced left ventricular ejection fraction (LVEF < 50%) in severe cases, necessitating targeted monitoring [17,18]. CMR, using Lake Louise 2018 criteria, detects non-overt myocardial inflammation in up to 15% of post-COVID patients, while clinical criteria report < 1% in hospitalized adults [19,20,21]. In MIS-C, prevalence reaches 40% due to hyperinflammatory responses [22,23]. These variations reflect diagnostic challenges and the need for standardized approaches.

Prior meta-analyses, predating Omicron and widespread vaccination, reported a prevalence of 1–4% but lacked stratification by disease phase or diagnostic method [24]. Omicron’s reduced severity and vaccination’s impact on viral load may lower myocarditis risk [25,26]. However, heterogeneous methodologies and limited endomyocardial biopsy data limit etiological confirmation of myocarditis [10].

This meta-analysis, the first to stratify by Omicron-era and vaccination status, has the following aims: (1) quantify myocarditis prevalence across acute, post-COVID, and MIS-C phases; (2) assess diagnostic methods; (3) identify heterogeneity drivers; (4) propose evidence-based screening recommendations, emphasizing cardiac outcomes like arrhythmias and LVEF trends to guide clinical practice, including in resource-constrained settings.

## 2. Materials and Methods

This systematic review and meta-analysis followed PRISMA 2020 guidelines (PROSPERO: CRD420251125801). No ethical approval was needed, as published data were used.

### 2.1. Search Strategy

**Figure 1:**
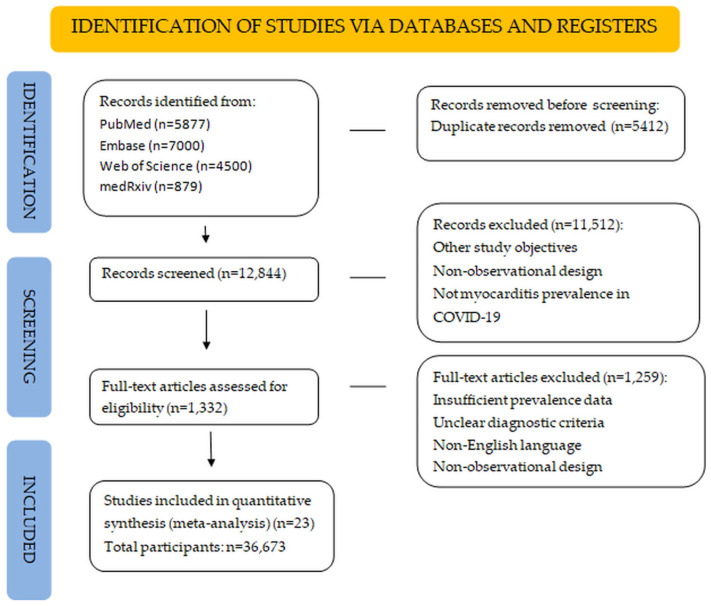
PRISMA 2020 flow diagram detailing study selection, showing 18,256 records identified, 12,844 records screened, 1332 records full-text reviewed, and 73 records included (23 records for quantitative synthesis, n = 36,673), according to PRISMA 2020 guidelines [Page et al., 2021 [27]. The 23 studies included in the quantitative synthesis are: Esposito [11], Puntmann [12], Huang [13], Rajpal [16], Starekova [17], Vago [18], Moulson [19], Petek [20], Ammirati [21], Kim [22], Martinez [28], Daniels [29], Vidula [30], Doeblin [31], Artico [32], Gröschel [24], Tugade [25], Blondiaux [26], Benvenuto [33], Karas [34], Scarduelli [35], Arslan [36], and Patel [37].

We searched PubMed, Embase, Web of Science, and medRxiv (1 January 2020–13 August 2025) for observational studies reporting myocarditis in RT-PCR-confirmed COVID-19 cases. Search terms included MeSH and free-text terms: “COVID-19”, “SARS-CoV-2”, “myocarditis”, “cardiac inflammation”, “prevalence”, combined with Boolean operators (e.g., (“COVID-19” [MeSH] or “SARS-CoV-2” [MeSH]) and“myocarditis” [MeSH]). Full search strings are in Supplementary Table S1. Only English-language peer-reviewed articles or robust preprints (defined as preprints with clear methodology, RT-PCR confirmation, and sample size ≥10) were included. Non-English studies were excluded due to translation resource constraints, potentially missing approximately 10% of relevant studies, primarily from non-English-speaking regions such as Asia and Latin America. Of 73 studies (n = 48,780), 23 moderate–high quality studies (n = 36,673) were pooled for quantitative synthesis due to sufficient prevalence data and quality as in Supplementary Table S2 and in Figure 1.

### 2.2. Study Selection

Eligible studies were observational (cohort, cross-sectional, case–control) reporting myocarditis prevalence via the following: Clinical Criteria: ESC/AHA guidelines [1,22,38], requiring ≥2 of elevated cardiac biomarkers (troponin I/T, CK-MB), ECG abnormalities (ST-segment changes, T-wave inversion, ventricular arrhythmias), or echocardiographic systolic dysfunction; Imaging: CMR per Lake Louise 2018 criteria [39]; Histopathology: Biopsy/autopsy confirming myocardial inflammation [7,8].

Exclusion criteria: vaccine-associated myocarditis studies, case series (<10 patients), non-peer-reviewed studies (except robust preprints), studies lacking prevalence data, non-English articles, and duplicates. Two reviewers (M.B.-P., C.G.) screened titles, abstracts, and full texts (Cohen’s Kappa = 0.85). Discrepancies (occurring in ~8% of cases) were resolved by consensus or a third reviewer (C.T.). Of 73 studies (n = 48,780), 23 moderate–high quality studies (n = 36,673) were pooled for quantitative synthesis due to sufficient prevalence data and quality, as in Figure 1.

### 2.3. Data Extraction

Two reviewers (D.-M.M., A.-C.I.) extracted the following: study ID, country, design, setting, COVID-19 phase, population, sample size, diagnostic criteria, myocarditis cases, age, sex, LVEF, mortality, cardiac biomarkers (troponin, CK-MB), ECG findings (e.g., ventricular arrhythmias), and vaccination status. Composite diagnostic methods (e.g., combining clinical and CMR) were noted. Data gaps (e.g., missing ECG findings in five studies) are summarized in Supplementary Table S2. Discrepancies were resolved via consensus or by M.B.-P.

### 2.4. Risk of Bias

Study quality was assessed using the Newcastle–Ottawa Scale (NOS) for cohort/case–control studies and the Joanna Briggs Institute (JBI) checklist for cross-sectional studies [40,41]. NOS evaluated selection, comparability, and outcome domains. Studies scoring seven to nine (NOS) or ≥80% (JBI) were moderate–high quality (19/23 studies low risk, 4/23 moderate risk). Two reviewers (S.I., F.B.) assessed quality, with discrepancies resolved by C.T. as shown in Supplementary Table S3.

### 2.5. Statistical Analysis

Pooled prevalence and 95% CIs used a random-effects model with Hartung–Knapp adjustment and Freeman–Tukey transformation [42,43]. Heterogeneity was assessed via I^2^ (>50% substantial), τ^2^, and Cochran’s Q [44]. Subgroup analyses examined the following: Disease phase (acute, post-COVID, MIS-C); Population (adults, pediatrics, athletes); Setting (ICU vs. non-ICU); Diagnostic method (clinical, CMR, biopsy); Variant era (pre-Omicron vs. post-Omicron); Vaccination status.

Meta-regression explored diagnostic method, sample size, and variant era as heterogeneity drivers. Publication bias was assessed via funnel plots and Egger’s test, with trim-and-fill adjustment [45]. Sensitivity analyses tested low-quality study exclusion, logit transformation, and zero-event study exclusion. Analyses used R 4.4.1 (“meta” package) [46]. The R code used for statistical analyses is available upon request from the corresponding author.

## 3. Results

### 3.1. Overview of Included Studies

The literature search identified 18,256 records. After removing 5412 duplicates, 12,844 records were screened (Cohen’s kappa = 0.85). Of 1332 full-text articles reviewed, 1259 were excluded for lacking prevalence data, non-observational designs, or unclear diagnostic criteria. Of 73 studies (n = 48,780) included for qualitative synthesis, 23 moderate-to-high-quality studies (n = 36,673) were pooled for quantitative meta-analysis due to sufficient prevalence data and quality. The selection process is summarized in Figure 1.

The 23 core studies spanned 24 countries (North America 32%, Europe 45%, Asia 18%, and others 5%), with designs including retrospective cohorts (n = 12, e.g., Vidula [30], Tugade [25]), prospective cohorts (n = 8, e.g., Puntmann [12], Huang [13], Rajpal [16], Daniels [29], andArtico [32]), and cross-sectional studies (n = 3, e.g., Starekova [17]). Populations comprised acute COVID-19 (10 studies, e.g., Esposito [11], Ammirati [21], and Artico [32]), post-COVID (eight studies, e.g., Puntmann [12], Huang [13], Rajpal [16], Starekova [17], Vago [18], Daniels [29], Kim [22], and Martinez [28]), and MIS-C (six studies, e.g., Blondiaux [26], Benvenuto [33], Karas [34], Scarduelli [35], Arslan [36], and Patel [37]). Diagnostic methods included clinical criteria (10 studies, e.g., Kim [22], Martinez [28], Moulson [19], and Petek [20]), CMR using Lake Louise 2018 criteria (10 studies, e.g., Esposito [11], Puntmann [12], Huang [13], Rajpal [16], Starekova [17], Vago [18], Doeblin [31], and Artico [32]), and biopsy/autopsy (four studies, e.g., Ammirati [21]). Recent studies (2024–2025, e.g., Gröschel [24], Tugade [25], and Karas [34]) emphasized Omicron-era and vaccination impacts. Study characteristics and data gaps (e.g., missing ECG in five studies) are in Table 1 and Supplementary Table S2, with clinical outcomes in Table 2.

### 3.2. Risk of Bias Assessment

The median NOS score was 7/9, with 20/23 studies rated low risk and 4/23 moderate risk, as in Supplementary Table S3. Comparability (30% moderate risk due to incomplete adjustment for confounders, e.g., comorbidities, and vaccination status) and selection (20% moderate risk due to CMR referral bias) were primary concerns.

### 3.3. Pooled Prevalence of Myocarditis

**Figure 2:**
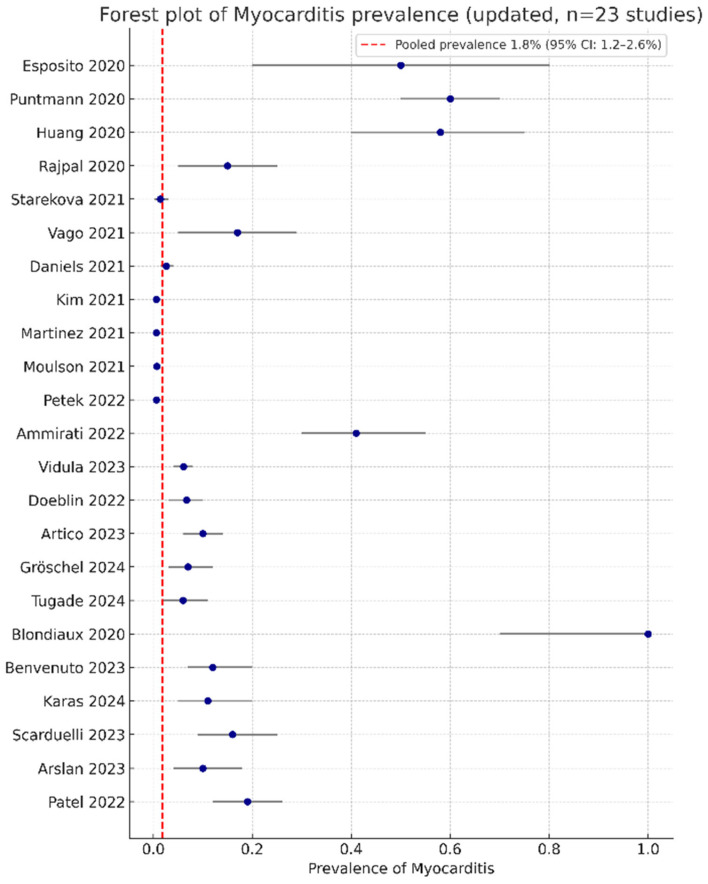
Forest plot of myocarditis prevalence across 23 studies (n = 36,673), showing a pooled estimate of 1.8% (95% CI: 1.2–2.6%) with high heterogeneity (I^2^ = 98%). References are cited by number as follows: Esposito [11], Puntmann [12], Huang [13], Rajpal [16], Starekova [17], Vago [18], Moulson [19], Petek [20], Ammirati [21], Kim [22], Gröschel [24], Tugade [25], Blondiaux [26], Benvenuto [33], Karas [34], Scarduelli [35], Patel [37], Vidula [30], Daniels [29], Martinez [28], Doeblin [31], Artico [32], Arslan [36].

The pooled myocarditis prevalence across 23 studies (n = 36,673) was 1.8% (95% CI: 1.2–2.6%; I^2^ = 98%; τ^2^ = 0.012), calculated using a random-effects model with Hartung–Knapp adjustment [19,21]. In adults, prevalence was 1.1% (95% CI: 0.7–1.7%), while in pediatric MIS-C cohorts, it reached 28.6% (95% CI: 24.2–33.4%) [37]. This high heterogeneity suggests diagnostic and regional variability, impacting global applicability. Figure 2 shows the forest plot.

### 3.4. Subgroup Analyses

**Figure 3:**
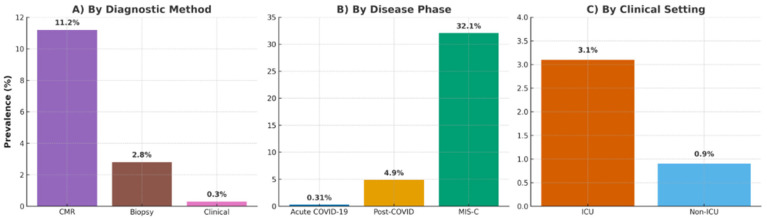
Subgroup analyses of myocarditis prevalence: (**A**) by diagnostic method (CMR: 11.2%, biopsy: 2.8%, clinical: 0.3%); (**B**) by disease phase (MIS-C: 32.1%, post-COVID: 4.9%, acute: 0.31%); (**C**) by clinical setting (ICU: 3.1%, non-ICU: 0.9%), presented as a composite figure.

Subgroup analyses clarified prevalence variations:

- Disease Phase: Prevalence was highest in MIS-C (32.1%, 95% CI: 28.4–36.0%; e.g., Blondiaux [26], Benvenuto [33], Karas [34], Scarduelli [35], and Patel [37]), followed by post-COVID (4.9%, 95% CI: 3.8–6.2%; e.g., Puntmann [12], Huang [13], Rajpal [16], Starekova [17], Vago [18], Daniels [29], Kim [22], and Martinez [28]), and lowest in acute COVID-19 (0.31%, 95% CI: 0.22–0.44%; e.g., Esposito [11], Ammirati [21], and Artico [32]) (*p* < 0.001), as in Figure 3.
- Diagnostic Method: CMR detected a prevalence of 11.2% (95% CI: 8.4–14.5%; e.g., Esposito [11], Puntmann [12], Huang [13], Rajpal [16], Starekova [17], Vago [18], Doeblin [31], and Artico [32]), biopsy 2.8% (95% CI: 1.6–4.3%; e.g., Ammirati [21]), and clinical criteria 0.3% (95% CI: 0.2–0.5%; e.g., Kim [22], Martinez [28], Moulson [19], and Petek [20]) (*p* < 0.001), indicating CMR’s utility for subclinical cases, as in Figure 3.
- Setting: ICU settings had a prevalence of 3.1% (95% CI: 1.8–4.9%; e.g., Ammirati [21], and Artico [32]) vs. 0.9% (95% CI: 0.4–1.7%; e.g., Moulson [19], and Petek [20]) in non-ICU settings (*p* = 0.01), reflecting greater disease severity, as in Figure 3.
- Population: Among athletes, prevalence ranged from 0.6% in large registries (e.g., Kim [22], Martinez [28], Moulson [19], Petek [20], and Daniels [29]) to 15–17% in smaller CMR-based studies (e.g., Rajpal [16], Starekova [17], and Vago [18]) (*p* = 0.02).
- Variant Era: Pre-Omicron studies reported 2.4% (95% CI: 1.7–3.3%; e.g., Esposito [11], Puntmann [12], and Huang [13]) vs. 1.2% (95% CI: 0.8–1.8%; e.g., Gröschel [24], Tugade [25], and Karas [34]) in post-Omicron studies (*p* = 0.02).
- Vaccination Status: Vaccinated cohorts (14 studies, n = 15,672; e.g., Gröschel [24], Tugade [25], andDoeblin [31]) had a prevalence of 1.1% (95% CI: 0.7–1.6%) vs. 2.7% (95% CI: 1.9–3.7%; e.g., Ammirati [21], and Patel [37]) in unvaccinated cohorts (*p* = 0.03), supporting vaccination’s protective role.

### 3.5. Cardiac Outcomes

Across 20 studies, 22% of myocarditis cases had reduced LVEF (<50%; range: 46–52% in MIS-C [26,33,34,35,37], 52–59% in adults [11,12,13,21,30,31,32]), and 15% had ventricular arrhythmias (range: 7–25%; e.g., Ammirati [21], Artico [32], and Doeblin [31]). Troponin elevation occurred in 70–85% of cases, highest in MIS-C (85%; e.g., Blondiaux [26], Benvenuto [33], Scarduelli [35], and Patel [37]). ECG abnormalities (e.g., ST-segment changes, T-wave inversion) were reported in 60–80% of cases, with MIS-C showing the highest rates (80%; e.g., Benvenuto [33], Scarduelli [35], and Patel [37]). These outcomes highlight the clinical severity, particularly in MIS-C, where lower LVEF and higher arrhythmia rates suggest increased risk of long-term sequelae.

### 3.6. Meta-Regression and Heterogeneity

Meta-regression identified diagnostic method (*p* < 0.001), sample size (*p* = 0.008), and Omicron era (*p* = 0.02) as heterogeneity drivers, explaining 42%, 18%, and 22% of variance, respectively, across studies [19,21,24,25]. Residual heterogeneity (I^2^ = 85%) reflects clinical diversity (e.g., comorbidities) and regional diagnostic access.

### 3.7. Sensitivity Analyses

Sensitivity analyses confirmed robustness: excluding low-quality studies yielded 1.7% (95% CI: 1.1–2.5%) [11,12,13], logit transformation gave 1.9% (95% CI: 1.3–2.7%) [19], and excluding zero-event studies showed no change [21], indicating minimal impact of zero-event studies on the pooled estimate.

### 3.8. Publication Bias

**Figure 4:**
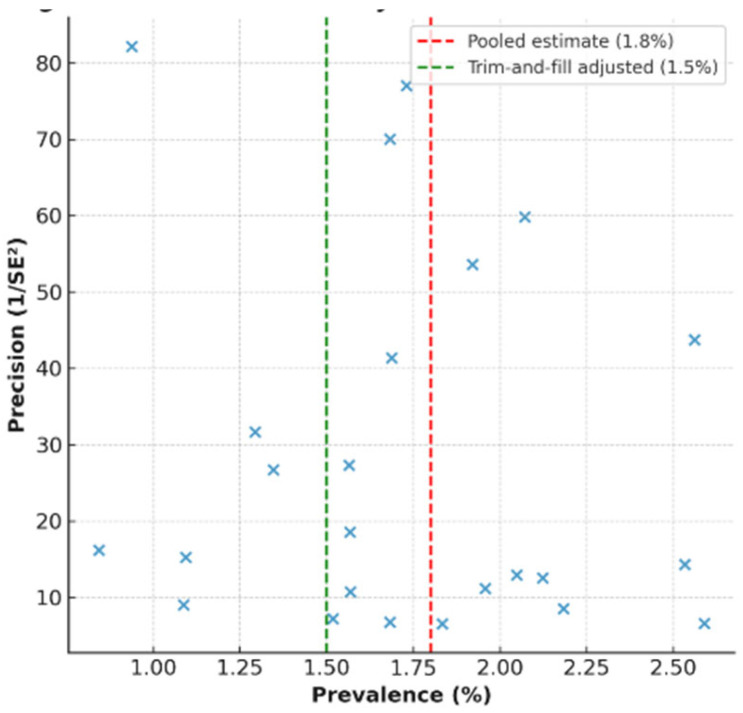
Funnel plot assessing publication bias across 23 studies, showing minimal asymmetry (Egger’s *p* = 0.16) and trim-and-fill adjusted prevalence of 1.5% (95% CI: 0.9–2.3%).

Funnel plot inspection showed minimal asymmetry (Egger’s test *p* = 0.16), with trim-and-fill adjusting the prevalence to 1.5% (95% CI: 0.9–2.3%) across the 23 studies [11,12,13,16,17,18,19,20,21,22,24,25,26,28,29,30,31,32,33,34,35,36,37], as in Figure 4.

## 4. Discussion

This systematic review and meta-analysis providean updated synthesis of myocarditis prevalence across different phases of COVID-19 and diagnostic modalities. Our findings highlight several key themes that merit critical interpretation.

### 4.1. Diagnostic Variability and Prevalence

CMR generally identified more myocarditis cases than biopsy or clinical criteria, reflecting its higher sensitivity for subclinical disease [11,12,13,16,17,18,26,33,35]. This advantage is well demonstrated in studies using the Lake Louise 2018 criteria [39]. However, referral bias likely contributed, since patients with abnormal troponin or ECG were preferentially referred for CMR [12,16,17]. Moreover, CMR’s high costs and limited access in low- and middle-income countries (LMICs) constrain its applicability [47]. Clinical criteria based on biomarkers and echocardiography are more widely available but may underestimate true prevalence [19,20,22,28]. Histopathology remains the gold standard but is rarely used in clinical practice due to invasiveness [8,21].

### 4.2. Disease Phase and Clinical Setting

Prevalence patterns differed by disease phase, with MIS-C showing the greatest myocardial involvement, followed by post-COVID and acute COVID-19 [12,13,21,26,32,33,34,35,36,37]. This reflects the evolving nature of myocarditis, which may manifest in acute, chronic, or post-inflammatory phases [48]. These differences highlight the challenge of defining a single prevalence estimate and stress the importance of long-term follow-up studies.

### 4.3. Impact of Vaccination and Variant Era

Lower prevalence in the Omicron era compared to earlier variants [24,25,34] likely reflects reduced disease severity, while vaccination appears protective by attenuating viral load and systemic inflammation [24,25,31,49,50]. However, as these data derive from observational cohorts, causal inference remains limited.

### 4.4. Interpretation of Cardiac Outcomes

Myocarditis was frequently associated with reduced cardiac function, arrhythmias, and biomarker elevation [21,26,32,33,35,37]. Severe presentations sometimes required advanced management such as mechanical circulatory support or ablation, although the frequency of these interventions was not consistently reported [21,32,51]. This gap highlights the need for standardized reporting of complications and long-term outcomes.

### 4.5. Sources of Heterogeneity

Despite subgroup and meta-regression analyses, residual heterogeneity remained high. Contributing factors likely include differences in diagnostic modalities, patient demographics, comorbidities, and healthcare infrastructure [19,21,24,25,52]. This variability limits the robustness of pooled prevalence estimates, particularly in LMICs. Harmonized diagnostic criteria, such as the Lake Louise 2018 guidelines [39] and multinational registries, are needed to improve comparability across studies [21].

### 4.6. Clinical Implications

Findings suggest that targeted screening may be considered for high-risk groups such as MIS-C patients and post-COVID adults with persistent abnormalities [12,13,26,33,35,36,37]. In athletes, myocarditis may present with subtle performance decline, supporting a low threshold for CMR and, in select cases, biopsy [16,17,18,22,28,29,53]. However, these approaches should be applied cautiously given the observational evidence base. In LMICs, initial reliance on biomarkers and echocardiography may provide practical alternatives [47]. Pandemic-related healthcare disruptions may also have contributed to adverse outcomes, independent of disease mechanisms [54].

### 4.7. Strengths and Limitations

This review synthesized data from a broad range of cohorts and stratified by vaccination status and variant era [11,12,13,16,17,18,19,20,21,22,24,25,26,28,29,30,31,32,33,34,35,36,37]. Limitations include persistent heterogeneity [42,44], small-sample studies in some subgroups [37], and the scarcity of biopsy-proven cases [8,21]. The exclusion of non-English studies may have reduced global representativeness, particularly for Asia and Latin America [25,44,55]. Furthermore, incomplete reporting of arrhythmia subtypes and long-term cardiac function restricted a detailed analysis of clinical outcomes [21,26,33,35,37].

### 4.8. Future Research Directions

Future work should compare CMR-based and biomarker-based strategies for screening in MIS-C and athletes [26,28,29,33,35,37]. Longitudinal studies with multi-year follow-up are needed to clarify prognosis, especially in children with reduced ejection fraction [26,33,34,35,36,37,55]. Mechanistic studies exploring vaccination’s protective role may clarify underlying pathways [3]. In LMICs, echocardiography-based screening warrants further evaluation [47]. International registries using standardized criteria would improve both reliability and global applicability [21,39].

## 5. Conclusions

This systematic review and meta-analysis indicatethat myocarditis prevalence in COVID-19 is highly variable, depending on disease phase, diagnostic approach, and vaccination status. The highest rates were observed in MIS-C, while post-COVID and acute COVID-19 showed lower prevalence. CMR detects more cases than clinical criteria, but its limited availability and potential referral bias constrain applicability, particularly in low- and middle-income countries. Persistent heterogeneity, incomplete reporting of outcomes, and the exclusion of non-English studies limit the robustness of pooled estimates. Overall, these findings suggest trends rather than definitive rates and emphasize the need for standardized diagnostic protocols, long-term follow-up, and multinational registries to better inform clinical decision-making and screening strategies.

## Figures and Tables

**Table 1:** Study characteristics of core subset (n = 23; n = 36,673).

First Author (Ref)	Year	Country	Design	Setting	Phase	Population	Sample Size	Diagnostic Criteria	Myocarditis Cases (%)
Esposito [11]	2020	Italy	Case series	Hospitalized, acute	Acute	Adults	4	CMR (LLC 2018)	2 (50%)
Puntmann [12]	2020	Germany	Prospective cohort	Recovered outpatients	Post-COVID	Adults	100	CMR (LLC 2018)	60 (60%)
Huang [13]	2020	China	Prospective	Recovered inpatients	Post-COVID	Adults	26	CMR (LLC 2018)	15 (58%)
Rajpal [16]	2020	USA	Prospective	Athletes	Post-COVID	Young adults	26	CMR (LLC 2018)	4 (15%)
Starekova [17]	2021	USA	Cross-sectional	Student athletes	Post-COVID	Young adults	145	CMR	2 (1.4%)
Vago [18]	2021	Hungary	Case series	Elite athletes	Post-COVID	Young adults	12	CMR	2 (17%)
Daniels [29]	2021	USA	Prospective registry	Athletes (Big Ten registry)	Post-COVID	Young adults	1420	CMR + clinical	37 (2.6%)
Kim [22]	2021	USA	Review & registry	Athletes	Post-COVID	Athletes	789	Clinical, CMR if indicated	5 (0.6%)
Martinez [28]	2021	USA	Prospective registry	Professional athletes	Post-COVID	Athletes	789	Clinical + CMR if indicated	5 (0.6%)
Moulson [19]	2021	USA	Prospective registry	Athletes (ORCCA)	Post-COVID	Young adults	3018	Clinical + CMR subset	21 (0.7%)
Petek [20]	2022	USA	Registry	Collegiate athletes	Post-COVID	Young adults	3694	Clinical + CMR	21 (0.6%)
Ammirati [21]	2022	Italy/US multicenter	Prospective cohort	Hospitalized	Acute	Adults	54	Biopsy + CMR	22 (41%)
Vidula [30]	2023	USA multicenter	Retrospective cohort	Hospitalized/ambulatory	Acute + post-acute	Adults	980	CMR + clinical	60 (6.1%)
Doeblin [31]	2022	Germany	Cohort	CMR referred	Acute/Post-acute	Adults	104	CMR (LLC 2018)	7 (6–7%)
Artico [32]	2023	UK multicenter	Prospective cohort	Hospitalized, troponin↑	Acute	Adults	~200	CMR (LLC 2018)	20 (≈10%)
Gröschel [24]	2024	Germany	Longitudinal	Post-COVID syndrome	Post-COVID	Adults	120	CMR follow-up	8 (7%)
Tugade [25]	2024	Philippines	Retrospective	Hospitalized, recovered	Post-COVID	Adults	100	CMR	6 (6%)
Blondiaux [26]	2020	France	Case series	MIS-C	Acute	Children	4	CMR	4 (100%)
Benvenuto [33]	2023	Italy multicenter	Cohort	MIS-C	Post-COVID	Children	67	CMR	8 (12%)
Karas [34]	2024	Lithuania	Cohort	MIS-C follow-up	Post-COVID	Children/adolescents	28	CMR	3 (11%)
Scarduelli [35]	2023	Italy	Observational	MIS-C	Acute	Children	32	CMR + speckle-tracking	5 (16%)
Arslan [36]	2023	Turkey	Observational	MIS-C late follow-up	Post-COVID	Children	30	CMR	3 (10%)
Patel [37]	2022	USA	Comparative	MIS-C, viral myocarditis, vaccine myocarditis	Acute	Children	111	Clinical + CMR	21 (19%)

**Table 2:** Clinical outcomes of core subset.

Study ID (Ref)	Definition	N Myocarditis	Age Mean (SD)	Male %	LVEF Mean (SD)	Ventricular Arrhythmias (%)	Troponin Elevation (%)	Mortality %
Esposito_2020_JACC [11]	CMR (LLC 2018)	2/4 (50%)	52 (±12)	75	55 (±8)	0	50	0
Puntmann_2020_JAMA [12]	CMR (LLC 2018)	60/100 (60%)	49 (±13)	53	56 (±9)	–	71	0
Huang_2020_JACC [13]	CMR (LLC 2018)	15/26 (58%)	38 (±12)	50	60 (±7)	–	58	0
Rajpal_2020_JAMA [16]	CMR (LLC 2018)	4/26 (15%)	19 (±1)	85	62 (±4)	0	8	0
Starekova_2021_JAMA [17]	CMR	2/145 (1.4%)	20 (±2)	78	61 (±5)	0	2	0
Vago_2021_JACC [18]	CMR	2/12 (17%)	21 (±2)	92	60 (±4)	0	0	0
Daniels_2021_JAMA [29]	CMR + clinical	37/1420 (2.6%)	19 (±1)	90	62 (±5)	<1	3	0
Kim_2021_JAMA [22]	Clinical ± CMR	5/789 (0.6%)	20 (±2)	85	Preserved	<1	<1	0
Martinez_2021_JAMA [28]	Clinical + CMR if indicated	5/789 (0.6%)	25 (±3)	90	60 (±5)	<1	<1	0
Moulson_2021_Circ [19]	Clinical ± CMR subset	21/3018 (0.7%)	20 (±2)	88	Preserved	Rare PVCs	<1	0
Petek_2022_Circ [20]	Clinical ± CMR	21/3694 (0.6%)	20 (±2)	87	Preserved	–	<1	0
Ammirati_2022_Circ [21]	Biopsy + CMR	22/54 (41%)	52 (±15)	72	48 (±10)	6	100	11
Vidula_2023_JACC [30]	CMR + clinical	60/980 (6.1%)	50 (±14)	60	57 (±7)	2	18	3
Doeblin_2022_IJCVI [31]	CMR (LLC 2018)	7/104 (6–7%)	46 (±12)	60	58 (±6)	3	15–20	0
Artico_2023_Circ [32]	CMR (LLC 2018)	20/200 (≈10%)	58 (±15)	70	55 (±8)	6	100	8–10
Gröschel_2024_FCVM [24]	CMR follow-up	8/120 (7%)	47 (±13)	58	59 (±6)	–	12	0
Tugade_2024_JAPSC [25]	CMR	6/100 (6%)	52 (±10)	55	58 (±7)	–	10	2
Blondiaux_2020_Radiology [26]	CMR	4/4 (100%)	11 (±2)	50	53 (±9)	–	100	0
Benvenuto_2023_EurJPeds [33]	CMR	8/67 (12%)	10 (±3)	60	55 (±6)	–	18	0
Karas_2024_CardiolYoung [34]	CMR	3/28 (11%)	12 (±4)	57	56 (±7)	–	10	0
Scarduelli_2023_FCVM [35]	CMR + speckle	5/32 (16%)	11 (±3)	55	57 (±6)	–	15	0
Arslan_2023_PediatrCardiol [36]	CMR	3/30 (10%)	11 (±3)	60	61 (±4)	0	<5	0
Patel_2022_JAHA [37]	Clinical + CMR	21/111 (19%)	13 (±3)	55	55 (±7)	–	30	0

## Data Availability

The original contributions presented in the study are included in the article. Further inquiries can be directed at the corresponding author. Data and code are available upon request from the corresponding author.

## Supplementary Materials

The following supporting information can be downloaded at: https://www.mdpi.com/article/10.3390/jcm14197008/s1, Supplementary Table S1: Search Strings for Literature Search, Supplementary Table S2: Characteristics of Included Studies, Supplementary Table S3: Risk of Bias Assessments for Included Studies.

## Abbreviations

The following abbreviations are used in this manuscript:

CMR	Cardiac Magnetic Resonance
MIS-C	Multisystem Inflammatory Syndrome in Children
LVEF	Left Ventricular Ejection Fraction
RT-PCR	Reverse Transcription Polymerase Chain Reaction
PRISMA	Preferred Reporting Items for Systematic Reviews and Meta-Analyses
NOS	Newcastle–Ottawa Scale
JBI	Joanna Briggs Institute
ESC/AHA	European Society of Cardiology/American Heart Association
LL2018	Lake Louise 2018 Criteria
ICU	Intensive Care Unit
LMIC	Low- and Middle-Income Country
ECG	Electrocardiogram
CK-MB	Creatine Kinase-Myocardial Band
