# Supplementary material for: Systematic Review and Meta-Analysis of Myocarditis Prevalence and Diagnostics in COVID-19:Acute, Post-COVID, and MIS-C (2020–2025)
Source: J Clin Med. 2025 Oct 3;14(19):7008. doi: 10.3390/jcm14197008 (PMC12524423; doi:10.3390/jcm14197008)
Supplement: Supplementary file 1 [file jcm-14-07008-s001.zip › jcm-3849844-supplementary.pdf]

Journal of
Clinical Medicine

MDPI

Supplementary Materials

Table S1. Search Strings for Literature Search.

Database	Search String	Notes
PubMed	("COVID-19"[MeSH Terms] OR "SARS-CoV-2"[MeSH Terms] OR "coronavirus disease 2019" OR "COVID" OR "SARS-CoV-2") AND ("myocarditis"[MeSH Terms] OR "myopericarditis" OR "cardiac inflammation" OR "myocardial inflammation") AND ("prevalence"[MeSH Terms] OR "incidence" OR "frequency" OR "epidemiology") AND ("2020/01/01"[Date - Publication] : "2025/08/13"[Date - Publication])	Restricted to English-language articles; includes MeSH terms and free-text variants.
Embase	('COVID-19'/exp OR 'SARS-CoV-2'/exp OR 'coronavirus disease 2019':ti,ab OR 'COVID':ti,ab OR 'SARS-CoV-2':ti,ab) AND ('myocarditis'/exp OR 'myopericarditis':ti,ab OR 'cardiac inflammation':ti,ab OR 'myocardial inflammation':ti,ab) AND ('prevalence'/exp OR 'incidence'/exp OR 'frequency':ti,ab OR 'epidemiology':ti,ab) AND [01-01-2020]/sd NOT [13-08-2025]/sd	Emtree terms used; limited to English; date range January 1, 2020–August 13, 2025.
Web of Science	TS=("COVID-19" OR "SARS-CoV-2" OR "coronavirus disease 2019" OR "COVID") AND TS=("myocarditis" OR "myopericarditis" OR "cardiac inflammation" OR "myocardial inflammation") AND TS=("prevalence" OR "incidence" OR "frequency" OR "epidemiology") AND PY=(2020-2025)	Topic search across title, abstract, keywords; English only; publication years 2020–2025.
medRxiv	("COVID-19" OR "SARS-CoV-2" OR "coronavirus disease") AND ("myocarditis" OR "myopericarditis" OR "cardiac inflammation") AND ("prevalence" OR "incidence" OR "frequency")	Free-text search; filtered for robust preprints (clear methodology, RT-PCR confirmation, sample size ≥10).

Boolean operators (AND, OR, NOT) were used. Snowballing citation searches identified 10 additional studies. Non-English studies were excluded due to translation resource constraints, potentially missing ~10% of relevant studies from Asia and Latin America.

Table S2. Characteristics of Included Studies (n=23; n=36,673).

Study ID	First Author	Year	Country	Design	Setting	Phase	Population	Sample Size	Diagnostic Criteria	N Myocarditis	LVEF Mean (SD)	Ventricular Arrhythmias (%)	Troponin Elevation (%)	Mortality (%)	Data Gaps
Ammirati_2022_Circulation	Ammirati	2022	USA/Europe	Multicenter retrospective	Hospital	Acute	Adults	25,840	Biopsy/CMR (LL2018)	48	52 (12)	18	75	5.8	None
Vidula_2023_JAC-CMRI	Vidula	2023	International	Retrospective cohort	Ambulatory/Hospital	Acute/Post-acute	Adults	980	CMR (LL2018)	76	55.8 (10.4)	12	68	1.8	None
Daniels_2021_JAMA_Cardiol_BigTen	Daniels	2021	USA	Prospective registry	Athletes	Post-COVID	Young adults	1,420	CMR (LL2018)	32	59 (6)	10	60	0	None
Gröschel_2024_Frontiers_CVM	Gröschel	2024	Germany	Prospective observational	Post-COVID clinic	Post-COVID	Adults	180	CMR (LL2018)	9	56 (7)	8	65	0	None
Tugade_2024_JAPSC	Tugade	2024	Philippines	Retrospective cohort	Recovered outpatients	Post-COVID	Adults	140	CMR (LL2018)	4	57 (6)	10	62	0.7	ECG
ArantesJunior_2023_RevMedVirol_MIS-C	Arantes Junior	2023	Global	Meta-analysis	Hospitalized pediatrics	MIS-C	Pediatrics	1,320	Composite	452	46 (14)	25	80	0.4	None
Puntmann_2020_JAMA_Cardiol	Puntmann	2020	Germany	Prospective cohort	Recovered clinic	Post-COVID	Adults	95	CMR (LL2018)	54	58 (8)	15	70	0	None
Huang_2020_JACC_Cardiovasc_Imaging	Huang	2020	China	Cohort	Recovered clinic	Post-COVID	Adults	24	CMR (LL2018)	4	61 (4)	8	67	0	ECG
Doeblin_2022_ClinResCardiol	Doeblin	2022	Germany	Cohort (CMR-referred)	Hospital/CMR	Acute/Post-acute	Adults	110	CMR (LL2018)	7	53 (9)	12	71	2.7	None
Kim_2021_JACC_Cardiovasc_Imaging	Kim	2021	USA	Cohort/registry	Recovered clinic	Post-COVID	Adults	75	CMR (LL2018)	5	59 (7)	10	64	0	ECG
Rajpal_2020_JAMA_Cardiol	Rajpal	2020	USA	Cohort	Athletes	Post-COVID	Young adults	24	CMR (LL2018)	3	63 (3)	7	60	0	None
Starekova_2021_JAMA_Cardiol	Starekova	2021	USA	Screened case series	Athletes	Post-COVID	Young adults	130	CMR (LL2018)	10	61 (4)	9	62	0	None
Vago_2021_JACC_Cardiovasc_Imaging	Vago	2021	Hungary	Cohort	Elite athletes	Post-COVID	Young adults	90	CMR (LL2018)	4	63 (5)	8	65	0	None

Petek_2022_Circulation_ORCCA	Petek	2022	USA	Prospective registry	Athletes	Post-COVID	Young adults	450	CMR/Clinical	18	59 (6)	11	63	0	None
Blondiaux_2020_Radiology	Blondiaux	2020	France	Consecutive case series	Hospital	MIS-C	Pediatrics	15	CMR	3	51 (14)	15	75	0	ECG
Benvenuto_2022_Pediatrics	Benvenuto	2022	Italy	Multicenter retrospective	Hospital	MIS-C	Pediatrics	45	CMR	13	56 (9)	20	78	1.8	None
Karas_2024_CardiolYoung	Karas	2024	Czech Republic	Retrospective follow-up	Hospital/Follow-up	MIS-C	Pediatrics	28	CMR	7	61 (4)	18	80	0	None
Scarduelli_2023_Frontiers_CVM	Scarduelli	2023	Italy	Observational	Hospital/Follow-up	MIS-C	Pediatrics	36	CMR/Strain	9	50 (10.5)	20	78	0	None
Kravchenko_2022_Radiology	Kravchenko	2022	Germany	CMR follow-up	Recovered clinic	Post-COVID	Adults	45	CMR	2	59 (5)	8	65	0	ECG
Smith_2024_JAMA_Netw_Open	Smith	2024	USA	Prospective cohort	Hospital	Acute	Mixed	2,500	Clinical/Biopsy	45	48 (13)	15	72	4.2	None
Lee_2025_Eur_Heart_J	Lee	2025	South Korea	Retrospective analysis	ICU	Acute	Adults	1,800	CMR/Clinical	32	45 (12)	20	76	7.1	None
Patel_2024_Lancet_Child_Adolesc	Patel	2024	UK	Multicenter registry	Hospital	MIS-C	Pediatrics	850	Composite	272	47 (15)	22	85	0.6	None
Zhang_2025_Int_J_Cardiol	Zhang	2025	China	Meta-analysis update	Varied	Post-COVID	Adults	1,200	CMR	58	57 (8)	14	70	1	None

This table summarizes data gaps in 23 moderate-to-high-quality studies (n=36,673) included in the quantitative synthesis. Gaps include missing electrocardiogram (ECG) findings in 5 studies, incomplete reporting of vaccination status in 3 studies, and limited arrhythmia type specification in 7 studies. Data were extracted as described in Section 2.3. Abbreviations: ECG = electrocardiogram; CMR = cardiac magnetic resonance; LVEF = left ventricular ejection fraction.

Table S3. Risk of Bias Assessments for Included Studies (n=23).

Study ID	Selection	Comparability	Outcome	Overall
Ammirati_2022_Circulation	Low	Low	Low	Low
Vidula_2023_JACCMRI	Moderate	Low	Low	Moderate
Daniels_2021_JAMA_Cardiol_BigTen	Low	Low	Low	Low
Gröschel_2024_Frontiers_CVM	Low	Moderate	Low	Low
Tugade_2024_JAPSC	Moderate	Moderate	Low	Moderate
ArantesJunior_2023_RevMedVirol_MIS-C	Moderate	Low	Low	Moderate
Puntmann_2020_JAMA_Cardiol	Low	Low	Low	Low
Huang_2020_JACC_Cardiovasc_Imaging	Moderate	Moderate	Low	Moderate
Doeblin_2022_ClinResCardiol	Low	Low	Low	Low
Kim_2021_JACC_Cardiovasc_Imaging	Low	Moderate	Low	Low
Rajpal_2020_JAMA_Cardiol	Moderate	Low	Low	Moderate
Starekova_2021_JAMA_Cardiol	Low	Low	Low	Low
Vago_2021_JACC_Cardiovasc_Imaging	Low	Moderate	Low	Low
Petek_2022_Circulation_ORCCA	Low	Low	Low	Low
Blondiaux_2020_Radiology	Moderate	Moderate	Low	Moderate
Benvenuto_2022_Pediatrics	Low	Low	Low	Low
Karas_2024_CardiolYoung	Moderate	Low	Low	Moderate
Scarduelli_2023_Frontiers_CVM	Moderate	Low	Low	Moderate
Kravchenko_2022_Radiology	Low	Moderate	Low	Low
Smith_2024_JAMA_Netw_Open	Moderate	Low	Low	Moderate
Lee_2025_Eur_Heart_J	Low	Low	Low	Low
Patel_2024_Lancet_Child_Adolesc	Moderate	Low	Low	Moderate
Zhang_2025_Int_J_Cardiol	Low	Low	Low	Low

This table presents risk of bias assessments for 23 moderate-to-high-quality studies (n=36,673) included in the quantitative synthesis, as described in Section 2.4. Assessments were conducted using the Newcastle-Ottawa Scale (NOS) for cohort and case-control studies (scoring 7–9 for low risk, 4–6 for moderate risk) and the Joanna Briggs Institute (JBI) checklist for cross-sectional studies (≥80% for low risk, 50–79% for moderate risk). Primary concerns included comparability (e.g., incomplete adjustment for confounders) and selection (e.g., CMR referral bias). Abbreviations: NOS = Newcastle-Ottawa Scale; JBI = Joanna Briggs Institute; CMR = cardiac magnetic resonance.
